# Genome-Wide Identification and Characterization of Growth Regulatory Factor Gene Family in *Helianthus annuus* and Functional Analysis of *HaGRF2c*

**DOI:** 10.3390/plants14223484

**Published:** 2025-11-14

**Authors:** Shiyu Yun, Xin Zhang

**Affiliations:** Institute of Industrial Crops, Shanxi Agricultural University, Taiyuan 030031, China

**Keywords:** growth regulating factor, *Helianthus annuus*, *HaGRF2c*, structure, function

## Abstract

Growth regulatory factors (GRFs) are sequence-specific DNA-binding transcription factors that play pivotal roles in regulating plant growth and development, and in enhancing plant tolerance to biotic and abiotic stresses. Although genome-wide structural and evolutionary studies have mapped and analyzed *GRF* genes in different plant species, knowledge of their characteristics and functions in sunflower (*Helianthus annuus*) remains limited. In this study, we used bioinformatics analyses and transgenic experiments to systematically analyze the structure and function of these genes. A total of 17 *HaGRF* genes were identified and classified into four distinct clades, with members of the same clade sharing conserved exon-intron structures and domain architectures. All HaGRFs were predicted to localize to the nucleus, which was experimentally verified for HaGRF2c, HaGRF3, and HaGRF8c. Transcriptome analysis demonstrated tissue-specific expression and stress-responsive profiles among the *HaGRF* genes. Quantitative real-time PCR revealed that several *HaGRF* genes were significantly induced under polyethylene glycol and NaCl stress. Additionally, ectopic expression of *HaGRF2c* in *Arabidopsis* enhanced growth and conferred greater drought tolerance, supporting its dual functions in regulating growth and in adapting to stress. In summary, this research elucidates the evolutionary relationships, conserved structural characteristics, expression patterns, and roles of the *HaGRF* gene family in sunflowers. These findings not only deepen our understanding of the biological functions of GRF transcription factors in sunflowers but also provide valuable candidate genes for improving yield and stress resistance in *H. annuus*.

## 1. Introduction

Growth regulatory factors (GRFs) are plant-specific transcription factors (TFs) widely distributed in plants, playing essential roles in regulating plant growth and development, and in mediating responses to abiotic stresses [1]. Structurally, GRF proteins exhibit distinct regional features. The N-terminus is highly conserved and contains two signature domains: the QLQ (QX3LX2Q) and WRC (CX9CX10CX2H) domains [2,3]. The QLQ domain interacts with the SNH domain of GRF-Interacting Factors (GIFs), which also possess a highly conserved N-terminal region, together participating in the regulation of downstream gene expression [4]. Meanwhile, the WRC domain includes a C3H DNA-binding motif and a nuclear localization signal (NLS) region that are critical for DNA binding and targeting of the TFs to the nucleus [5]. In contrast to the conserved N-terminal region, the C-terminal region of GRFs varies in length and amino acid composition. This region contains several motifs with low conservation, such as TQL (Thr, Gln, Leu), FFD (Phe, Phe, Asp), and GGPL (Gly, Gly, Pro, Leu) [6]. This structural heterogeneity contributes directly to variations in the size and functional diversity of GRF proteins [4,5,6]. Despite its low sequence similarity, the C-terminal region is indispensable for transcriptional activation. For example, AtGRF proteins (*Arabidopsis*) with a truncated C-terminal region, OsGRF10 (*Oryza sativa*), and ZmGRF10 (*Zea mays*) with short C-terminal regions lose their transactivation activities [4,7,8].

The first GRF, OsGRF1, was identified in deep-water rice in 2000 and has been shown to regulate in gibberellin (GA)-induced stem elongation [2]. The advent of high-throughput gene sequencing technologies has enabled the genome-wide identification and characterization of the GRF family in various plant species. To date, nine GRF members have been identified in *Arabidopsis* [3], 12 in *O. sativa* [5], 17 in *Z. mays* [9], 30 in *Triticum aestivum* [10], 10 in *Saccharum spontaneum* [11], 16 in *Medicago sativa* [12], 24 in *Zanthoxylum armatum* [13], 8 in *Nelumbo nucifera* [14], 22 in *Glycine max* [15], 24 in *Arachis hypogaea* [16], 13 in *Solanum lycopersicum* [17], and 9 in *Vitis vinifera* [18].

Functional studies have demonstrated that GRFs play crucial roles in many aspects of plant growth and development, including flowering, cell proliferation, stem elongation, and seed development. In *O. sativa*, RNA interference silencing of *OsGRF3*, *OsGRF4*, and *OsGRF5* leads to dwarfing, retarded growth, and delayed inflorescence formation [19]. *OsGRF1/2/3/7/8/10/12* are implicated in the regulation of GA-induced stem elongation [5]. In *Arabidopsis*, *AtGRF1*/*2*/*3*/*4*/*5*/*9* regulate leaf shape and size through cell proliferation [20,21,22,23]. Overexpression of *AtGRF4*, *AtGRF5*, and *AtGRF6* represses the *KNOTTED1*-*LIKE HOMEOBOX* (*KNOX*) gene, which is associated with organ formation and shoot apical meristem differentiation [19,24]. Additionally, 35S:*AtGRF5* promotes chloroplast division and photosynthesis [25]. In *Z. mays*, *ZmGRF*10 overexpression decreases leaf size and plant height [8]. While differential expression of *MsGRF* genes in *M. sativa* suggests a role in leaf size regulation [26]. In *Brassica napus*, *BnGRF2* enhances seed oil production by regulating cell number and photosynthesis [27]. Beyond growth regulation, GRFs are involved in abiotic stress responses. For instance, A missense mutation in *OsGRF4* improves transgenic plant performance under cold stress [28]. *AtGRF7* in *Arabidopsis* binds to the promoter of *dehydration-responsive element*-*binding protein 2A* (*DREB2A*), repressing its expression and modulating osmotic stress response [29]. Similarly, *VvGRF7* (*V. vinifera*) overexpression increases growth and enhances osmotic stress sensitivity in transgenic *Arabidopsis* plants [18]. Additionally, *MsGRF2* and *MsGRF6* in *M. sativa* are significantly upregulated under osmotic stress [12], while *GhGRF1a*-*At* and *GhGRF9b*-*Dt* in *Gossypium hirsutum* show decreased expression under cold and polyethylene glycol (PEG) stresses, but are upregulated under heat and salt stress [30]. Collectively, *GRFs* are multifunctional regulators that orchestrate plant growth and development, and play critical roles in mediating tolerance to diverse abiotic stresses.

Sunflower (*Helianthus annuus* L.) is an annual species in the family Asteraceae, with over 70 known species worldwide. As the fourth most important oil crop globally, sunflower is also valued as an ornamental plant [31]. It is a significant source of premium oil and dietary fiber for human consumption [32,33], and sunflower meal is used in livestock feed to improve animal growth and productivity [34,35]. However, sunflowers face numerous abiotic and biotic stressors during their lifecycle, with drought being one of the most significant factors affecting growth and yield [36]. GRFs are involved not only in plant growth and reproduction but also in responses to these stressors. Therefore, investigating the roles of GRFs in sunflowers is important for advancing crop genetics and improving yield and quality.

This study was conducted to identify and characterize the *GRF* gene family in sunflower (*HaGRF*) through whole-genome analysis, including gene family identification, phylogenetic tree analysis, gene structure analysis, subcellular localization, chromosome mapping, and gene replication studies. Additionally, the expression profiles of these genes were examined across nine different tissues and their responses to NaCl, PEG, and various hormonal stresses. These findings provide a basis for future research into the functional roles of *HaGRF* genes.

## 2. Results

### 2.1. Identification of GRF Genes

Seventeen *HaGRF* genes were identified within the sunflower genome, designated *HaGRF1a* to *HaGRF9*. Analysis of their coding sequences (CDS) revealed considerable variation in length, with the shortest being 468 bp in *HaGRF9* and the longest reaching 1527 bp in *HaGRF2b*. These corresponded to predicted protein sizes ranging from 155 to 508 amino acids (aa). Predictions of physicochemical properties showed that molecular weights (MWs) of HaGRF proteins varied between 17.27 kDa (HaGRF9) and 55.48 kDa (HaGRF2b). The isoelectric points (pI) values ranged from 6.40 for HaGRF8c to 9.66 for HaGRF3. Most HaGRF proteins had pI values greater than 7 were weakly alkaline, with HaGRF8c being the only weakly acidic member (pI < 7). The differences in aa sequence lengths and pI among the GRFs indicate variation in aa number or proportion, which may contribute to the functional diversity of the *GRF* family. All HaGRF proteins were classified as hydrophilic proteins based on their negative grand average of hydropathicity (GRAVY) scores.

Subcellular localization predictions indicated that all HaGRFs were in the nucleus. Comparative structural analysis of *HaGRF* and *AtGRF* genes revealed intron numbers ranging from one to five, with most members containing two to three introns. Specifically, *HaGRF1b* and *HaGRF9* had only one intron, while *AtGRF8* had five (Figure S1). The corresponding exon numbers ranged from two (*HaGRF1b*, *HaGRF9*) to five (*HaGRF5c*, *HaGRF6a*, and *HaGRF6b*; Table 1).

### 2.2. Phylogenetic Analysis

**Figure 1:**
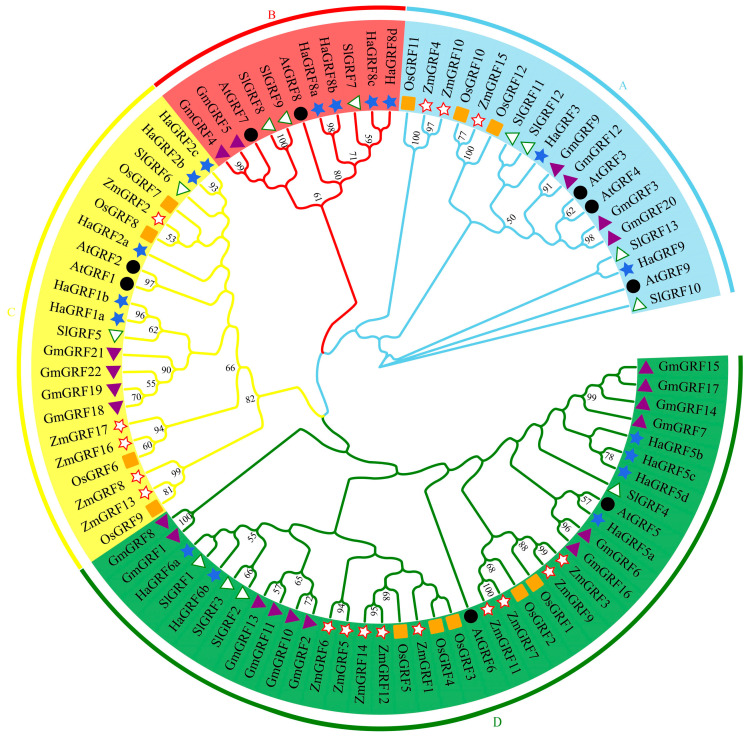
Phylogenetic analysis of the GRF protein family in six plant species. Bootstrap support values are labeled at the branch nodes. The GRF proteins were classified into four clades (clade A–D), distinguished by different background colors. Clade A (sky blue), Clade B (red), Clade C (yellow), and Clade D (green). Different species are represented by distinct symbols. Hollow circles represent AtGRF, orange rectangles represent OsGRF, green and white triangles represent SlGRF, red and white stars represent ZmGRF, purple triangles represent GmGRF, and royal blue stars represent HaGRF.

To explore the evolutionary relationships between HaGRF proteins and their homologs in other species, a phylogenetic tree was constructed using 73 GRF protein sequences. This dataset included predicted HaGRF proteins as well as previously reported GRF proteins from *Arabidopsis* (9), *O. sativa* (12), *Z. mays* (17), *G. max* (22), and *S. lycopersicum* (13). The accession numbers for these *GRF* gene IDs are provided in Table S1. The analysis revealed that the 73 GRF proteins were grouped into four distinct clades (A–D). Among these, clade D was the largest, containing 38 members from all six plant species, including six HaGRFs—the highest number of HaGRFs found in any clade. In contrast, clade B was the smallest, comprising four HaGRFs, and included GRFs from *S. lycopersicum*, *Arabidopsis*, *G. max*, and *H. annuus*, suggesting that this clade may be dicot-specific. Clades A and C contained two and five HaGRFs, respectively (Figure 1). Further phylogenetic analysis showed that most HaGRFs were closely related to GRFs from dicot species, such as SlGRF, AtGRF, and GmGRF, and were more distantly related to monocot GRFs, including ZmGRF and OsGRF. These findings indicate that HaGRFs are more closely related to dicot GRF proteins than to monocot GRF proteins

### 2.3. Gene Structure and Motif Analysis

**Figure 2:**
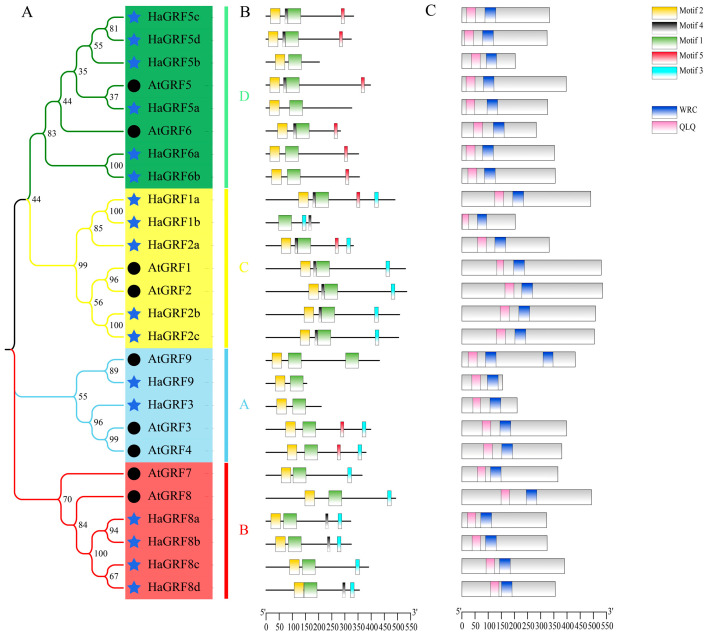
Evolutionary relationships and structural analysis of HaGRFs and AtGRFs. (**A**): Phylogenetic tree of HaGRF and AtGRF proteins. Different background colors and branch colors represent distinct clades of the HaGRF and AtGRF families. Hollow circles represent AtGRF and royal blue stars represent HaGRF. (**B**): Conserved motif analysis. (**C**): Conserved domain analysis. Boxes of different colors indicate distinct motifs or domains. The scale bars at the bottom of the figure indicate the length of the aa sequence.

**Figure 3:**
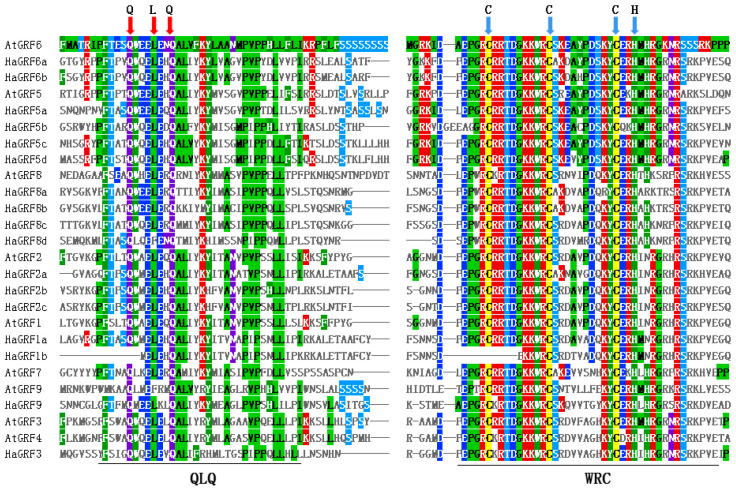
Multiple sequence alignment of the HaGRF and AtGRF aa sequences. Based on the results of protein sequence alignment, Mview classifies and colors amino acids according to their physicochemical properties such as polarity, charge, and hydrophobicity. QLQ and WRC domains are indicated at the bottom. QLQ (red arrows) and CCCH (blue arrows) residues are marked at the top of the alignment to highlight conserved functional sites.

Domain analysis using the Conserved Domain Database (CDD) revealed that all HaGRF and AtGRF proteins contained QLQ and WRC domains in their N-terminal regions, excluding AtGRF9, which possessed two WRC domains (Figure 2C). Multiple sequence alignment confirmed that the WRC domain generally contained a zinc-finger structure (Cys-Cys-Cys-His, CCCH), although HaGRF1b lacked the first C residue. The N-terminal QLQ domain generally featured a conserved QLQ (Gln-Leu-Gln) structure. However, HaGRF1b lacked the first Q residue, whereas HaGRF8d and AtGRF9 exhibited an L-to-F aa substitution (Figure 3). In addition, five conserved motifs were identified. Motifs 1 and 2, which corresponded to the WRC and QLQ domains, were detected in most HaGRF and AtGRF proteins. Notably, unlike the domain analysis, HaGRF1b lacked motif 2 (QLQ), likely because the first Q residue was absent in its QLQ sequence. Motifs 3 and 5, corresponding to GGPL and FFD, respectively, were found in certain GRF family members. GRFs within the same clade displayed similar motif compositions: all members of clades B and C contained motif 3, while clade B proteins lacked motif 5, and clade A did not contain motif 4. (Figure 2B). All the motif sequences are presented in Table S2. In summary, GRFs from the same clade exhibited conserved motifs and domains, suggesting that both sequence features and functional characteristics were evolutionarily conserved within the same clade.

### 2.4. Secondary Structure and Three-Dimensional Structure

Secondary structure prediction revealed that all HaGRF and AtGRF proteins comprise four fundamental secondary structural elements: α-helix, extended chain, β-turn, and random coil. Both HaGRF and AtGRF families displayed similar compositional trends, with random coils comprising the largest portion of the secondary structure—averaging 64.42% in *H. annuus* and 64.23% in *Arabidopsis*. Alpha-helices were the next most prevalent, accounting for 13.24–39.35% in HaGRFs and 12.64–24.62% in AtGRFs. Extended strands contributed 6.19% to 18.23%, while β-turns represented the smallest proportion, ranging from 1.12% to 9.68% (Table S3).

**Figure 4:**
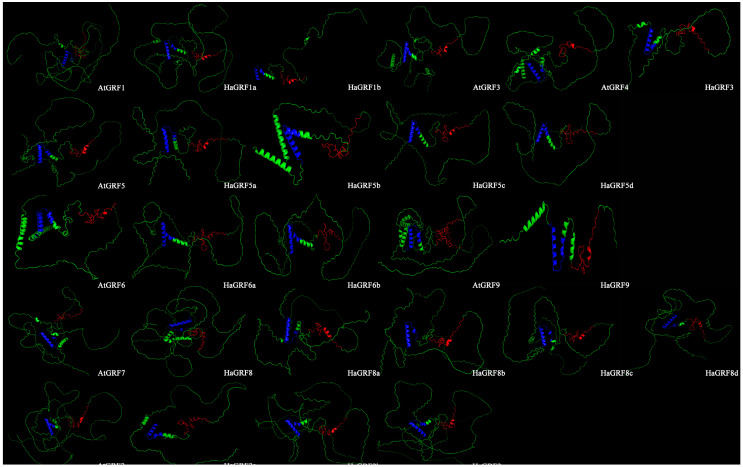
Three-dimensional structure predictions of HaGRFs and AtGRFs. The amino acid backbone is colored green. The WRC domain is displayed in red, and the QLQ domain in blue to distinguish core functional domains.

Further examination of the three-dimensional (3D) structures of HaGRF and AtGRF revealed a similar architecture, lacking intricate spiral folding. The 3D structural results corroborated the secondary structure predictions, showing significant spatial similarity between the HaGRF and AtGRF proteins. Notably, the QLQ domain was characterized by two distinct α-helices, while the WRC domain predominantly comprised random coils (Figure 4). This alignment between secondary and 3D structures was observed across all predicted proteins within the same clade, highlighting a high degree of structural consistency.

### 2.5. Chromosome Location and Collinearity Analysis

**Figure 5:**
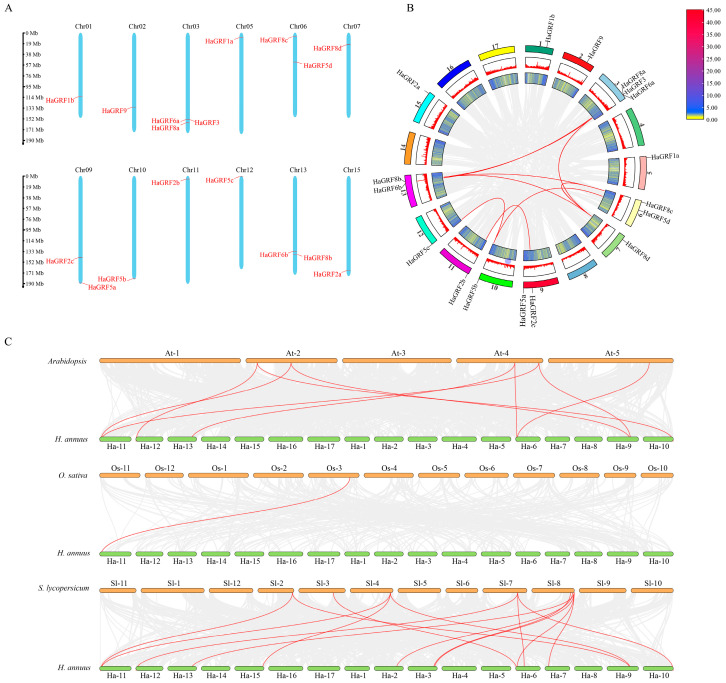
Chromosomal localization, intragenomic collinearity, and interspecific synteny of *HaGRF* genes. (**A**) Chromosomal localization of *HaGRF* genes: left scale bar indicates chromosome length (unit: Mb), chromosome numbers are labeled at the top of each bar. (**B**) Intragenomic collinearity of *HaGRF* genes. (**C**) Interspecific synteny of *GRF* genes between *HaGRF* and three other *GRF* gene families. Duplicated *GRF* gene pairs are highlighted with red curves.

Genome annotation revealed that the 17 identified *HaGRF* genes were distributed across 12 *H. annuus* chromosomes with uneven distribution. Chromosomes 01, 02, 05, 07, 10, 11, 12, and 15 contained one *HaGRF* gene each, while chromosomes 06, 09, and 13 contained two genes; chromosome 03 exhibited the highest density, hosting three *HaGRF* genes. Notably, most *HaGRF* genes were mainly distributed at the chromosome ends (Figure 5A).

To elucidate gene duplication relationships within the *HaGRF* family, collinearity analysis was performed using TBtools-II v2.323. All *HaGRFs* were found to be derived from duplication events with distinct duplication types: 12 genes (70.6%) were labeled as whole-genome repeats (WGD) or segmental repeats, four genes (23.6%) were classified as dispersed repeats, and only *HaGRF6a* was recognized as a proximal duplication gene. No tandem duplication events were detected. In other species, *GRFs* in *Arabidopsis* were predominantly classified as WGD or segmental duplication genes (66.7%), while the remainder were dispersed duplications (33.3%). In *O. sativa*, a strong bias toward dispersed duplication was noted, with 91.7% (11 of 12) of *OsGRF* genes in this category. In *S. lycopersicum*, WGD or segmental duplication genes accounted for 61.5%, and dispersed genes accounted for 38.5%. These findings suggest that WGD or segmental duplication and dispersed duplication are the primary drivers of *GRF* gene family expansion (Table S4).

Internal collinearity analysis in sunflowers indicated that nine pairs of *GRF* genes (11 genes) were located within collinearity blocks (Figure 5B). Comparative syntenic mapping between *HaGRF* and *GRF* gene families in *Arabidopsis*, *O. sativa*, and *S. lycopersicum* was performed to illustrate phylogenetic relationships. Syntenic analysis revealed 9 orthologous gene pairs between *HaGRF* and *AtGRF*, 1 between *HaGRF* and *OsGRF*, and 15 between *HaGRF* and *SlGRF* (Figure 5C). The abundance of collinear gene pairs indicated strong collinearity between HaGRF and related species on the phylogenetic tree. Notably, *HaGRF2b* formed orthologous pairs with *AtGRF1*, *AtGRF2*, *OsGRF6*, *SlGRF5*, and *SlGRF6*, suggesting a potentially important evolutionary role for *HaGRF2b* within the *GRF* family. To further assess evolutionary selection pressure during *GRF* gene family formation, Ka/Ks ratios were calculated for all gene pairs. All Ka/Ks values were less than 1, indicating that the *GRF* gene family underwent purifying selection during evolution (Table S5).

### 2.6. Gene Ontology (GO) Annotation

GO annotation analysis was conducted to explore the potential biological functions of HaGRF proteins. The results indicated that HaGRF proteins were involved in diverse biological, cellular, and molecular processes. Most notably, the predominant HaGRF proteins were enriched in the regulation of transcription, i.e., the DNA-template process (GO: 0006355). Fifteen of the 17 HaGRFs were further annotated to participate in the developmental process (GO: 0032502), with HaGRF5a and HaGRF5b specifically implicated in leaf development (GO: 0048366), underscoring their pivotal role in the growth and development of *H. annuus*. Regarding molecular function, HaGRF proteins were primarily associated with ATP binding (GO: 0005524), which could provide the necessary energy for DNA binding and protein–protein interactions. Cellular localization analysis revealed that all HaGRF proteins were localized in the nucleus (GO: 0005634), and HaGRF5a was also found in the membrane (GO: 0016021) (Table S6). These results were consistent with the predicted subcellular localization outcomes presented in Table 1.

### 2.7. Analysis of the Cis-Acting Element of GRF Genes

Cis-acting elements are crucial regulatory sequences that influence the binding of TFs, thereby controlling the expression of downstream target genes. In this study, a comprehensive analysis was conducted on the 1.5 kb promoter regions of *HaGRF* genes to identify and classify cis-acting elements based on their functional roles. These elements were grouped into four major categories: hormone response elements, stress and defense response elements, growth and development-related response elements, and light response elements.

**Figure 6:**
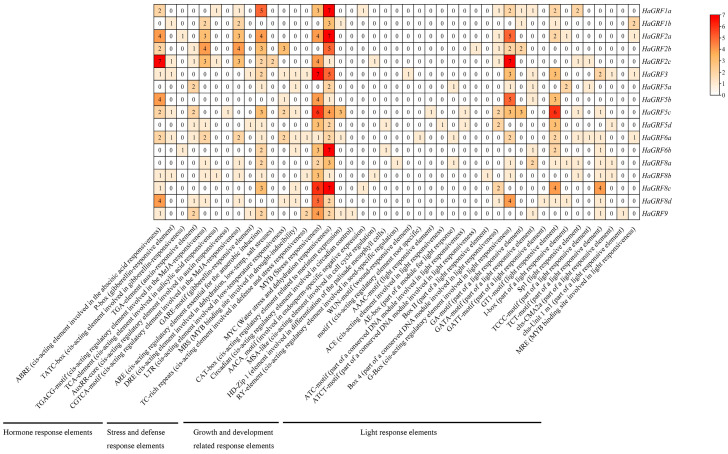
Cis-acting element analysis of the *HaGRF* gene promoters. Promoter regions (1.5 kb) of *HaGRF* genes were analyzed using the PlantCARE database. The heatmap presents the number of each cis-acting element in individual *HaGRF* promoters.

Hormone response elements identified include motifs associated with methyl jasmonate (MeJA)-responsiveness (CGTCA and TGACG motifs), auxin responsiveness (AuxRR core and TGA element), abscisic acid response (ABRE), salicylic acid (SA) response (TCA element), and gibberellin responsive elements (GARE motif, P-box, and TATC box). Stress and defense response elements primarily consisted of those involved in the low-temperature response (LTR), defense and stress response (TC-rich repeats), dehydration, low-temperature, and salt stress response (DRE), stress response (MYB), water stress and dehydration response (MYC). Notably, all *HaGRF* genes contained the MYC motif, while the DRE element was exclusively found in *HaGRF2c*. Elements related to growth and development included those regulating meristematic tissue expression (CAT-box), circadian rhythm control (Circadian), cell cycle regulation (MSA-like), and seed-specific regulation (RY-element). These elements were unevenly distributed, with some present only in specific *HaGRFs*, such as the Circadian element in *HaGRF9*, the RY element in *HaGRF8a*, and the WUN motif/wool-responsive element in *HaGRF3*, suggesting functional divergence among *GRF* genes. Light response elements were highly diverse and widely distributed throughout the promoter regions. Among these, the G-box and GT1 motifs were the most prevalent. Importantly, every *HaGRF* contained at least one hormone-related cis-acting element and one stress-related cis-acting element; however, the specific types varied considerably among individual genes (Figure 6 and Table S7).

### 2.8. Expression Patterns of HaGRFs in Various Tissues and Treatments

**Figure 7:**
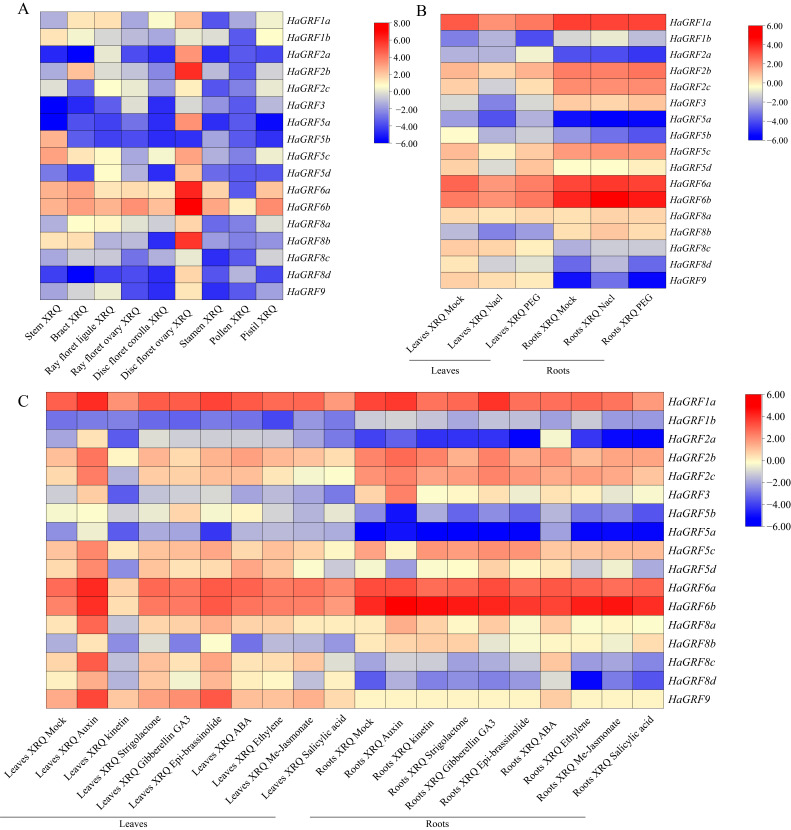
Tissue-specific and stress/hormone-induced expression profiles of *HaGRF* genes. (**A**) Tissue-specific expression patterns of *HaGRF* genes in nine sunflower tissues. (**B**) Expression profiles of *HaGRF* genes in sunflower leaves and roots under NaCl and PEG treatments. (**C**) Expression profiles of *HaGRF* genes in sunflower leaves and roots under treatments with nine hormones. All heatmaps were generated using TBtools-II v2.323 software. The red color indicates higher expression; the blue color indicates lower expression.

To investigate the expression profiles of *HaGRF* genes, transcriptomic analyses utilizing RNA-seq data were performed across nine distinct sunflower tissues: bract, stem, stamen, pistil, pollen, ray floret (RF) ligule, RF ovary, disc floret (DF) corolla, and DF ovary. Among these tissues, *HaGRF6b* exhibited the highest expression, whereas *HaGRF2a*, *HaGRF3,* and *HaGRF5b* maintained consistently low expression levels in most tissues. Distinct tissue-specific expression patterns were observed, with most *HaGRF*s showing higher expression levels in the DF ovary than in other tissues. Additionally, *HaGRF1b*, *HaGRF5b*, and *HaGRF5c* were highly expressed in the stem, and *HaGRF1a* was preferentially expressed in the RF ligament. The lowest overall expression levels were detected in pollen, suggesting a limited role for HaGRFs in pollen development and function (Figure 7A).

Expression responses of *HaGRF*s under abiotic stress conditions were also examined. RNA-seq analysis revealed that most *HaGRF*s were inhibited in leaves subjected to PEG and salt stress, while approximately half of the *HaGRF*s in roots were upregulated and the remainder were downregulated (Figure 7B).

The influence of hormonal treatments on *HaGRF* expression was assessed by analyzing expression levels in leaves and roots exposed to nine hormones: auxin, kinetin, strigolactone, gibberellic acid 3 (GA3), epi-brassinolide, abscisic acid (ABA), ethylene, MeJA, and SA. Under normal growth conditions, *HaGRF1a* exhibited the highest expression in leaves and roots, while *HaGRF5a* was the lowest. Auxin treatment generally induced moderate upregulation of most *HaGRFs* in both tissues. In contrast, MeJA and SA caused moderate downregulation, and several *HaGRF*s exhibited high expression in response to kinetin in leaves. These patterns indicate that *HaGRF* gene expression is dynamically regulated by various hormones and is involved in the sunflower hormone signaling pathway (Figure 7C).

**Figure 8:**
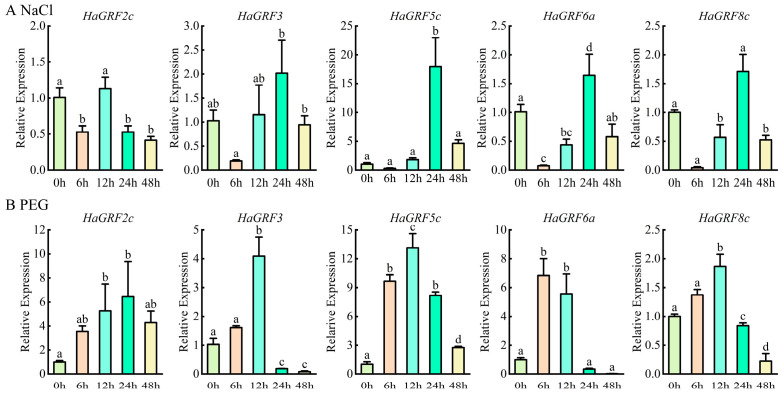
qRT-PCR analysis of *HaGRF* gene expression in sunflower leaves under NaCl (**A**) and PEG stress (**B**). Data are presented as the mean ± standard deviation (SD) from three independent biological replicates (*n* = 3). Significant differences relative to the 0 h control are marked with different lowercase letters (*p* < 0.05).

To further clarify the role of *HaGRF*s in abiotic stress response, five *HaGRF* genes were selected for quantitative real-time PCR (qRT-PCR) analysis of their expression in leaves under PEG and NaCl stress. All the selected *HaGRFs* exhibited overlapping but distinct expression patterns under the two treatments. They almost increased by at least two-fold at the 12 h time point under PEG stress, increased at the 24 h time point under NaCl stress, and then gradually decreased thereafter. These findings suggest that *HaGRF* genes exhibit stress-specific expression patterns and may play regulatory roles in sunflower responses to PEG and NaCl stress (Figure 8).

### 2.9. Subcellular Localization of HaGRF Proteins

**Figure 9:**
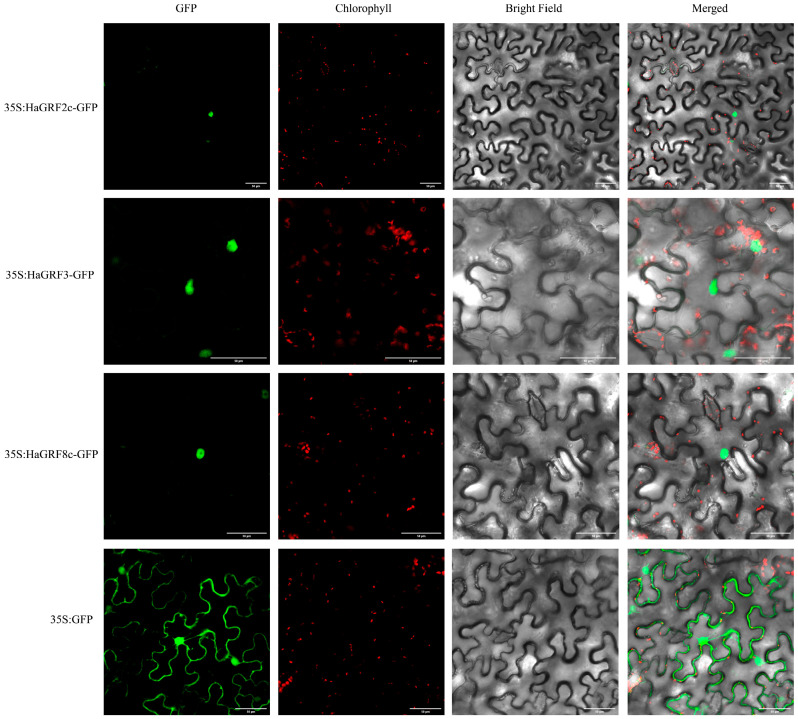
Subcellular location of HaGRF2c, HaGRF3, and HaGRF8c proteins in tobacco leaf epidermal cells. Fluorescence images were captured under four channels: green fluorescent protein (GFP) fluorescence (green), chlorophyll fluorescence (red), bright light, and merged light. Leaves were cultured under low light for 48 h before imaging with a laser confocal scanning microscope (LCSM). Scale bar = 50 μm.

To determine the subcellular localization of HaGRF proteins, three genes—*HaGRF2c*, *HaGRF3*, and *HaGRF8c*—were selected for analysis. These genes were fused with green fluorescent protein (GFP) expression vectors and transiently transformed into tobacco leaf epidermal cells. The GFP fluorescence signals from the recombinant constructs (35S:HaGRF2c:GFP, 35S:HaGRF3:GFP, and 35S:HaGRF8c:GFP) were exclusively detected in the nucleus. This localization confirmed that these HaGRF proteins were nuclear-localized (Figure 9), consistent with sequence-based predictions (Table 1).

### 2.10. Phenotypic Analysis of Arabidopsis

**Figure 10:**
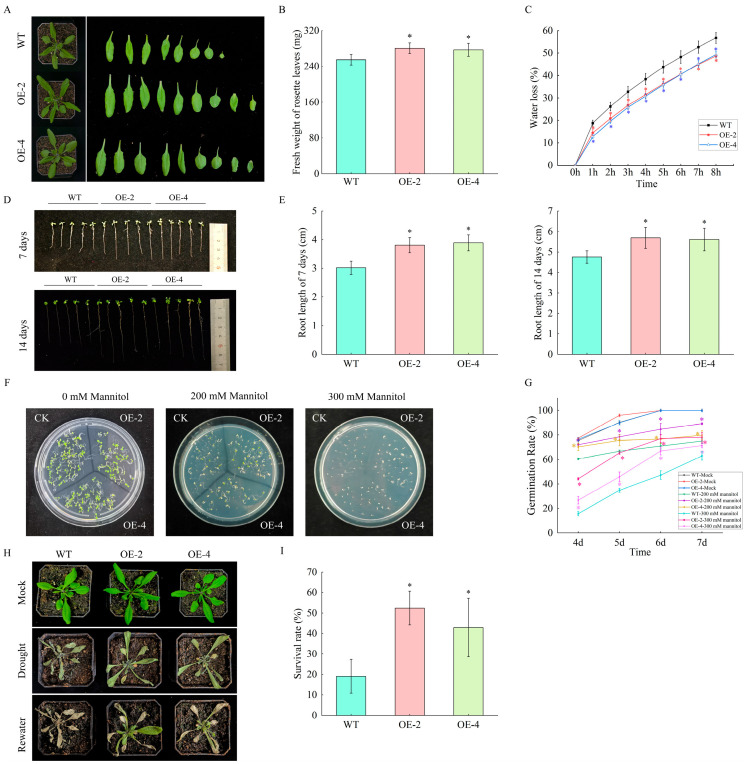
Functional characterization of *HaGRF2c* via ectopic expression in *Arabidopsis*. (**A**,**B**) Phenotypes (**A**) and fresh weight of rosette leaves (**B**) of 3-week-old WT and OE-2, OE-4 grown in a greenhouse. Data are presented as mean ± SD (*n* = 10). (**C**) Water loss rate in leaves of WT, OE-2 and OE-4 plants. Detached leaves were weighed at the indicated time points; data are mean ± SD (*n* = 5). (**D**,**E**) Phenotypes (**D**) and primary root lengths (**E**) of WT, OE-2, and OE-4 seedlings grown on 1/2 MS medium for one and two weeks. Data are mean ± SD (*n* = 10). (**F**,**G**) Germination phenotypes (**F**) and rates (**G**) of WT, OE-2, and OE-4 seeds grown on 1/2 MS medium supplemented with 0, 200, or 300 mM mannitol for 7 days. Each strain was tested with at least 25 seeds per culture medium, and the experiment was conducted with three independent biological replicates. (**H**,**I**) Phenotypic performance (**H**) and survival rates (**I**) of WT, OE-2, and OE-4 plants after exposure to drought stress for 10 days of drought stress and a 3-day rewatering recovery period. Each treatment included seven plants per line with three biological replicates. Significant differences relative to the WT are marked with “*” (*p* < 0.05).

To validate the functional roles of *HaGRFs* in plant growth and drought stress responses, transgenic *Arabidopsis* plants constitutively expressing *HaGRF2c* were generated. Two T3 transgenic *Arabidopsis* lines, OE-2 and OE-4, were selected for subsequent experiments, with wild-type (WT) *Arabidopsis* serving as the control. Sterilized T3 transgenic lines and WT *Arabidopsis* seeds were grown in a greenhouse for three weeks. The overexpression of *HaGRF2c* in *Arabidopsis* led to enhanced growth, as evidenced by a greater fresh weight (FW) of rosette leaves in transgenic plants compared to WT controls (Figure 10A,B). Leaf water loss was assessed by detaching rosette leaves from 3-week-old plants and measuring their weight hourly over 8 h. The transgenic lines OE-2 and OE-4 exhibited significantly lower leaf water loss rates than WT (Figure 10C). Seeds from WT, OE-2, and OE-4 were germinated on 1/2 MS medium for one and two weeks. Both OE-2 and OE-4 displayed increased root growth, with longer primary roots than WT seedlings (Figure 10D,E).

To assess drought tolerance, seeds from WT and transgenic lines were germinated on 1/2 MS medium supplemented with 200 mM or 300 mM mannitol and incubated in a controlled growth chamber (22 ± 2 °C, 16 h light/8 h dark photoperiod) for one week. The germination rates of the two transgenic lines were statistically higher than WT under both mannitol concentrations (Figure 10F,G). For the soil drought stress assay, 17-day-old WT and transgenic seedlings pre-cultured under well-watered conditions were subjected to drought stress by withholding water for 10 days. WT plants exhibited more pronounced yellowing and wilting compared to OE-2 and OE-4 overexpression lines (Figure 10H). After 3 days of rehydration, the survival rate of WT plants was approximately 20%, whereas the survival rates of OE-2 and OE-4 were approximately 45% (Figure 10I). Taken together, these results demonstrate that overexpression of *HaGRF2c* in *Arabidopsis* not only promotes growth but also confers enhanced tolerance to drought stress.

## 3. Discussion

The GRFs are a group of plant-specific TFs that play a significant role in regulating plant growth and development. Although the functions of *GRF* gene families have been extensively characterized in various plant species, systematic research focusing on *GRF* genes in *H. annuus* has been limited. This study undertook a comprehensive genome-wide identification and analysis of the *GRF* gene family in *H. annuus*, aiming to provide insights into their evolutionary features, structural attributes, and potential functional roles.

In this study, 17 *HaGRF* genes were identified in sunflower, exceeding the number in *Arabidopsis.* This increase may be attributed to two additional rounds of genome-wide duplication events during sunflower evolution [37]. The exon-intron structure is a key evolutionary feature, and its gain or loss often results in functional differences [38]. Analysis revealed that closely related gene pairs within the phylogenetic tree showed high similarity in aa length and exon-intron numbers. All *HaGRF* genes contained one to three introns and two to five exons (Figure S1). This structural feature is consistent with that of *BdGRFs* in *Brachypodium distachyon*, which also have one to three introns [39]. Furthermore, all HaGRF members possessed conserved QLQ and WRC domains in their N-terminal regions (Figure 2C), similar to GFRs from *O. sativa*, *Arabidopsis*, and *G. max* [2,3,40]. Notably, mutations in the QLQ domain residues of HaGRF1b and HaGRF8d may alter their protein interaction activities (Figure 3). Moreover, all HaGRF proteins were found to contain the WRC motif. TQL and GGPL motifs identified in the C-terminal region, which are also found in GRFs from other plant species (Figure 2B) [41,42]. These findings indicate that GRF proteins are structurally conserved; observed differences may contribute to functional diversification within the family.

All GRF family members were grouped into four distinct clades (Figure 1). Most HaGRFs clustered with SlGRFs, AtGRFs, and GmGRFs, likely due to their shared classification as dicotyledonous plants. GRFs from monocot species were predominantly found in three clades, with none in Clade B, suggesting a possible whole-genome triplication event in the ancestor of eudicots [18], that may have driven differentiation between monocot and dicot GRFs [43]. A recent report identified that a single *GRF* gene is present in the charophyte, *Klebsormidium nitens* [44], indicating that *GRFs* originated in streptophytes and expanded through genome duplication and retention [45]. Gene replication is a major evolutionary force, enabling plants to adapt and diversify [26]. Collinear analysis revealed nine orthologous gene pairs in *HaGRFs* (Figure 5B), suggesting duplication events during the *HaGRF* evolution. Gene duplication is a common event in plant evolutionary processes. Duplication types showed that *HaGRFs, SlGRFs*, and *AtGRFs* were predominantly WGD or segmental and dispersed duplicates (Table S4). This indicated that such duplication events have been the main forces driving the expansion of the *GRF* gene family [11,26,46]. Similar duplication patterns have been observed in other sunflower gene families, such as *HaAP2/ERF* [47], *HaJAZ* [31], and *HaWOX* [48], which have undergone WGD or segmental duplication events, further indicating that these duplication events are crucial for sunflower genome evolution. Homologous gene pair analysis revealed closer genetic relationships between *HaGRF* and *SlGRF/AtGRF* than between *HaGRF* and *OsGRF* (Figure 5C), further supporting the structural and functional differentiation between monocot and dicot GRFs. Ka/Ks ratio analysis indicated strong purifying selection pressure on *HaGRFs* (Table S5), similar to findings in other species, including *S. spontaneum*, *Actinidia chinensis*, and *M. sativa* [11,26,49], highlighting purifying selection as a conserved force that maintains the functional integrity of *GRF* genes across plant species.

Cis-acting elements in gene promoters provide binding sites for downstream gene regulation. *GRF* gene families are involved in various hormone signal transduction pathways, such as the brassinosteroid pathway in *Arabidopsis* and GA biosynthesis in *Nicotiana tabacum* [50]. In the current study, several hormone-related cis-acting elements, including those for GA3, MeJA, ABA, and SA, were identified in *HaGRF* promoters, suggesting involvement in multiple hormone pathways. The ABRE-binding proteins/factors (AREBs/ABFs) can activate *DREB2A* transcription via ABRE in response to osmotic stress. *AtGRF7* can bind to the *DREB2A* promoter to repress its expression, inhibit osmotic stress, and prevent growth inhibition [29]. *HaGRF8b/8c/8d*, which belongs to the clade B like *AtGRF7* (Figure 2A), also contains ABRE cis-acting elements that are speculated to be related to osmotic stress. Numerous light response elements in the promoters of *HaGRF* genes were also detected, indicating that *HaGRFs* may participate in light responsiveness and photosynthesis. Additionally, GRF responses to various abiotic stresses [12,18,29,51]. Each *HaGRF* gene contains abiotic stress-related cis-acting elements (Figure 6), suggesting roles in stress responses and providing candidates for further research on abiotic stress resistance.

Since gene expression patterns can reflect gene function, analysis of gene expression patterns revealed that certain *HaGRF* family members are highly expressed across diverse sunflower tissues, suggesting their involvement in regulating growth and development. Additionally, *HaGRF* genes exhibited significant induction under NaCl and PEG stress (Figure 8B), suggesting a potential role as early-responsive genes to salt and drought stress. Tissue- and stress-specific expression patterns may result from functionally distinct cis-acting elements in each gene, consistent with previous findings that *GRF* genes mediate plant responses to various abiotic stresses. Similar differential expression patterns have been reported for *StGRFs* in *S*. *tuberosum* under heat, salt, drought, and hormone treatment [52]. *LuGRF3*, *LuGRF12*, and *LuGRF16 in L. usitatissimum* are enriched in response to salt stress [53]. Several *GRF* genes from the *Populus P*ag*GRF11*, *Liriodendron chinense LcGRF2*, and *Arabidopsis AtGRF2*, homologs of the *HaGRF2c* gene, promote leaf development, leading to enlarged leaves and cotyledons [3,54,55]. Similarly, overexpression of *HaGRF2c* in *Arabidopsis* enhances growth and increases germination and survival rates under drought conditions (Figure 10). Notably, studies of *GRF* genes from other species have revealed functional differences in their response to drought stress. *CsGRF04*-VIGS (homologous to *AtGRF9*) lines exhibit significantly increased resistance to drought stress [56], whereas ectopic expression of *VvGRF7* (homologous to *AtGRF7*) promotes the growth and sensitivity of transgenic *Arabidopsis* plants to osmotic stress [18]. These functional differences may be due to the existence of distinct regulatory networks in different plants.

## 4. Materials and Methods

### 4.1. Identification of GRF Gene Family

The gene and protein sequences of the *AtGRF* gene family were retrieved from the TAIR database (http://www.Arabidopsis.org/, accessed on 10 March 2024). Using the *AtGRF* gene sequence as a template, homologous *GRF* genes in sunflower were identified via BLAST searches against the XRQr2.0 Genome Portal of INRAE Sunflower Bioinformatics Resources (https://www.heliagene.org/, accessed on 15 March 2024). Corresponding *HaGRF* gene and protein sequences were then downloaded for subsequent analysis. Candidate HaGRF proteins were validated using SMART/PFAM (http://smart.embl-heidelberg.de/, accessed on 18 March 2024) to confirm the presence of conserved QLQ and WRC domains; sequences lacking these domains were excluded from further analysis. The physicochemical properties of the HaGRF proteins were predicted using the ExPasy ProtParam tool (https://web.expasy.org/protparam/, accessed on 20 March 2024). Subcellular localization of HaGRF proteins was predicted via the Plant-mPLoc server (Predicting subcellular localization of plant proteins including those with multiple sites, http://www.csbio.sjtu.edu.cn/bioinf/plant-multi/, accessed on 15 March 2024). Gene structure, including intron numbers, was analyzed using GSDS2.0 (Gene Structure Display Server, http://gsds.gao-lab.org/, accessed on 20 March 2024) by comparing their CDSs with the corresponding genome sequences.

### 4.2. Phylogenetic Sequence and Structures Analysis

To clarify the phylogenetic relationships of GRF proteins across plant species, a phylogenetic tree was constructed using GRF amino acid sequences from six species: *H. annuus*, *S. lycopersicum*, *Z. mays*, *Arabidopsis*, *O. sativa*, and *G. max*. GRF protein sequences for different species were acquired from public databases: OsGRF and SlGRF sequences were downloaded from the Ensemble Plants database (https://plants.ensembl.org/index.html, accessed on 17 March 2024), while the ZmGRF and GmGRF sequences were downloaded from phytozome 13 (https://phytozome-next.jgi.doe.gov/blast-search, accessed on 22 March 2024). The phylogenetic tree was constructed using the neighbor-joining (NJ) method in MEGA 7 software with 1000 bootstrap replicates. The online software EvolView (https://evolgenius.info/evolview-v2/, accessed on 25 March 2024) was used to decorate the phylogenetic tree. Finally, based on phylogenetic relationships, HaGRF proteins were named as HaGRF1a to HaGRF9.

The phylogenetic relationships, conserved motifs, and secondary structures of HaGRF and AtGRF proteins were systematically analyzed in this study. Conserved motifs were identified by the Simple MEME Wrapper in TBtools-II v2.323 with parameters set as follows: the motif number of GRF set as 5, the width range of 6 to 50 amino acids (aa), Max E-value of 10, Mode set as Zero or One Occur Per Seq. Phylogenetic tree analysis was performed by MEGA7.0 with the same parameters as described above. Conserved Domain Database (CDD, https://www.ncbi.nlm.nih.gov/cdd/?term=, accessed on 3 April 2024) in NCBI was used to analyze the secondary structure. Additionally, Gene Structure View (Advanced) in the TBtools-II v2.323 software was used to visualize the above results.

### 4.3. Multiple Sequence Alignment and Analyses of Cis-Acting Elements

Multiple sequence alignment (MSA) of the amino acid sequences of HaGRF and AtGRF proteins was performed by Clustal Omega (https://www.ebi.ac.uk/jdispatcher/msa/clustalo, accessed on 5 April 2024), and visualized by Mview (A multiple alignment viewer, https://www.ebi.ac.uk/Tools/msa/mview/, accessed on 5 April 2024).

Cis-acting elements in *HaGRF* and *AtGRF* gene promoters were analyzed using PlantCARE (http://bioinformatics.psb.ugent.be/webtools/plantcare/html/, accessed on 8 April 2024) with 1.5 kb upstream sequences of each gene.

### 4.4. Chromosome Location and Collinearity Analysis

Based on the information in Table 1, the chromosomal location of *HaGRF* genes was mapped using TBtools-II v2.323. Relevant gene sequences and annotation information for *H. annuus*, *S. lycopersicum*, *Arabidopsis* and *O. sativa* were retrieved from the Ensemble Plants database (https://plants.ensembl.org/index.html, accessed on 10 April 2024). Both intraspecific synteny relationships (*H. annuus*) and interspecific synteny relationships (*H. annuus*, and *S. lycopersicum*, *Arabidopsis*, *O. sativa*) were analyzed and visualized by TBtools-II v2.323. The software was used to calculate the non-synonymous substitution rates (Ka) and synonymous substitution rates (Ks) of *GRF* gene pairs, with the Ka/Ks ratio used to assess the selection pressure.

### 4.5. Secondary Structure Prediction, Three-Dimensional Structure Modeling and Validation

Using the SOPMA website (https://npsa-prabi.ibcp.fr/, accessed on 9 April 2024) to predict the secondary structure of HaGRF and AtGRF proteins. Three-dimensional protein structures were modeled using SWISS-MODEL (https://beta.swissmodel.expasy.org, accessed on 12 April 2024) and visualized with PyMOL (https://pymol.org/2/, accessed on 17 April 2024).

### 4.6. Analysis of Expression Patterns

The tissue-specific expression patterns of *HaGRF* genes were analyzed using transcriptome data from the sunflower expression atlas, including multiple tissue types: stem, bract, ray floret ligule, ray floret ovary, disc floret corolla, disc floret ovary, stamen, pollen, and pistil. Additionally, expression profiles under NaCl, PEG, and hormonal treatments in leaves and roots were also analyzed, and heatmaps illustrating the gene expression levels were generated and visualized using TBtools-II v2.323.

### 4.7. Plant Cultivation and Treatment, RNA Extraction and qRT-PCR Analysis

The seeds of *H. annuus* were germinated in potting soil in a growth chamber under a controlled environment at 28 °C day/20 °C night, with a 16 h light/8 h dark photoperiod. For treatments, 28-day-old plants were sprayed with 200 mM NaCl and 20% PEG solution. Leaves were collected at 0, 6, 12, 24 and 48 h post-treatment for further expression analyses. All plant tissues were frozen in liquid nitrogen immediately after collection and stored at −80 °C until RNA extraction. Total RNA was extracted, and qRT-PCR was performed using SuperReal PreMix Plus (SYBR Green, TIANGEN, Beijing, China) following the manufacturer’s protocol. Each treatment was biologically replicated at least three times. The primer sequences are listed in Table S8.

### 4.8. Subcellular Localization

The cDNA of *HaGRF* was inserted into the modified pCambia1300 vector downstream of the 35S promoter and transformed into *Agrobacterium tumefaciens* strain GV3101. HaGRF proteins were transiently expressed as green fluorescent protein (GFP) fusion proteins in tobacco leaf epidermal cells. Two days after infiltration, the GFP fluorescence signal and chlorophyll fluorescence signal were observed with a laser confocal microscope (A1 HD25, Nikon, Tokyo, Japan) under excitation at 488 nm, 640 nm, respectively. Leaves transformed with the 35S-GFP vector alone served as positive controls.

### 4.9. Arabidopsis Transformation and Phenotypic Analysis

*HaGRF2c*-overexpressing constructs were introduced into the *Arabidopsis* using the floral-dip method. The transgenic lines were selected based on hygromycin B resistance (50 μg/mL) and verified by RT-PCR; the primer sequences are listed in Table S8. Homozygous T3 transgenic lines were obtained through successive screening on 1/2 Murashige and Skoog (MS) medium supplemented with 50 μg/mL hygromycin B, and used for subsequent phenotypic analyses.

### 4.10. Statistical Analysis

In the expression analysis, the values are presented as mean ± standard deviation (SD). The statistical result was analyzed with the ANOVA method followed by Duncan’s test using SPSS software 25, and *p* < 0.05 was considered to be significant.

## 5. Conclusions

In this study, we performed a genome-wide identification of the *GRF* gene family in *H. annuus* and identified 17 *HaGRF* genes. They clustered into four distinct clades; members within the same clade exhibited high similarity in amino acid sequences, gene structures, conserved domains, and motifs. Additionally, most *HaGRF* genes exhibited tissue-specific expression patterns and were induced by salt, drought and multiple hormonal stresses. Overexpression of *HaGRF2c* in *Arabidopsis* confirmed the dual-functional role of *HaGRF2c* in regulating growth and drought response. This study will provide new insights into the evolutionary relationship and functions of *HaGRF2c* in sunflower, and may provide a useful resource for future molecular breeding of drought-tolerant sunflowers.

## Figures and Tables

**Table 1:** Properties of the predicted HaGRF proteins.

Assigned Name	Primary Transcript ID	Locus	CDS Length (bp)	Peptide Length (aa)	Exon	Description	MW (kDa)	pI	GRAVY	Predicted Location
HaGRF1a	HanXRQr2_Chr05g0196271	HanXRQChr05:8466274…8468729 (+strand)	1473	490	4	growth-regulating factor 1-like	53.03	8.41	−0.524	Nucleus.
HaGRF1b	HanXRQr2_Chr01g0028371	HanXRQChr01:112730233…112731062 (−strand)	612	203	2	growth-regulating factor 6-like isoform X1	22.28	9.16	−0.495	Nucleus.
HaGRF2a	HanXRQr2_Chr15g0718821	HanXRQChr15:166533660…166535306 (+strand)	999	332	3	growth-regulating factor 6-like isoform X2	36.79	9.36	−0.518	Nucleus.
HaGRF2b	HanXRQr2_Chr11g0469341	HanXRQChr11:5164810…5170793 (−strand)	1527	508	4	growth-regulating factor 1-like	55.48	7.07	−0.725	Nucleus.
HaGRF2c	HanXRQr2_Chr09g0395961	HanXRQChr09:143380431…143382973	1515	504	4	growth-regulating factor 1-like	54.50	8.45	−0.674	Nucleus.
HaGRF3	HanXRQr2_Chr03g0125511	HanXRQChr03:153401166…153402344 (+strand)	633	210	3	growth-regulating factor 3-like	23.03	9.66	−0.596	Nucleus.
HaGRF5a	HanXRQr2_Chr09g0417451	HanXRQChr09:188622395…188623697 (+strand)	981	326	3	growth-regulating factor 5-like isoform X1	36.79	8.51	−0.913	Nucleus.
HaGRF5b	HanXRQr2_Chr10g0465971	HanXRQChr10:180813249…180814319 (−strand)	612	203	4	growth-regulating factor 1-like	23.47	9.49	−1.073	Nucleus.
HaGRF5c	HanXRQr2_Chr12g0521141	HanXRQChr12:615913…618507 (+strand)	1002	333	5	growth-regulating factor 1-like	38.10	8.17	−0.995	Nucleus.
HaGRF5d	HanXRQr2_Chr06g0260061	HanXRQChr06:51431400…51434352 (+strand)	975	324	4	growth-regulating factor 1-like isoform X2	37.16	7.71	−0.999	Nucleus.
HaGRF6a	HanXRQr2_Chr03g0125451	HanXRQChr03:153354173…153357709 (+strand)	1059	352	5	growth-regulating factor 4-like isoform X1	39.10	8.58	−0.770	Nucleus.
HaGRF6b	HanXRQr2_Chr13g0600651	HanXRQChr13:132413949…132420019 (+strand)	1068	355	5	growth-regulating factor 4-like isoform X1	39.18	8.63	−0.762	Nucleus.
HaGRF8a	HanXRQr2_Chr03g0127921	HanXRQChr03:158653418…158654909 (−strand)	969	322	4	growth-regulating factor 8-like	35.16	8.38	−0.777	Nucleus.
HaGRF8b	HanXRQr2_Chr13g0603261	HanXRQChr13:138108859…138111078 (−strand)	975	324	3	growth-regulating factor 8-like	35.63	8.04	−0.767	Nucleus.
HaGRF8c	HanXRQr2_Chr06g0242221	HanXRQChr06:6231651…6234272 (+strand)	1173	390	4	growth-regulating factor 8-like	42.81	6.40	−0.845	Nucleus.
HaGRF8d	HanXRQr2_Chr07g0284291	HanXRQChr07:20999552…21001489 (+strand)	1068	355	4	growth-regulating factor 8-like	39.06	7.59	−0.718	Nucleus.
HaGRF9	HanXRQr2_Chr02g0071791	HanXRQChr02:131329426…131330368 (−strand)	468	155	2	growth-regulating factor 10-like	17.27	8.67	−0.467	Nucleus.

## Data Availability

All data generated or analyzed during this study are included in this published article and its Supplementary Information Files.

## Supplementary Materials

The following supporting information can be downloaded at: https://www.mdpi.com/article/10.3390/plants14223484/s1, Table S1. Accession numbers of *GRF* gene IDs of *Arabidopsis*, *O. sativa*, *S. lycopersicum*, *Z. mays*, and *G. max*. Table S2. Motif consensus of HaGRFs. Table S3. Secondary structures for the HaGRFs and AtGRFs. Table S4. Duplication types in *GRFs*. Table S5. Ka and Ks values for the duplicated gene pairs. Table S6. Gene ontology annotations of the HaGRFs. Table S7. Distributions of cis-acting elements in all *HaGRFs.* Table S8. Sequences of primers. Figure S1. The gene structures of *HaGRFs* and *AtGRFs*. Gene structures are visualized with the following components: upstream/downstream regulatory regions (blue boxes), coding sequences (CDSs, yellow boxes), and introns (black lines). The phase of each intron (0, 1, or 2) is labeled above the corresponding intron. The scale bar at the bottom of the figure indicates the length of genetic elements (unit: bp) for uniform reference.
