# Supplementary figures and images for: Genome-Wide Identification and Characterization of Growth Regulatory Factor Gene Family in Helianthus annuus and Functional Analysis of HaGRF2c
Source: Plants (Basel). 2025 Nov 14;14(22):3484. doi: 10.3390/plants14223484 (PMC12656601; doi:10.3390/plants14223484)

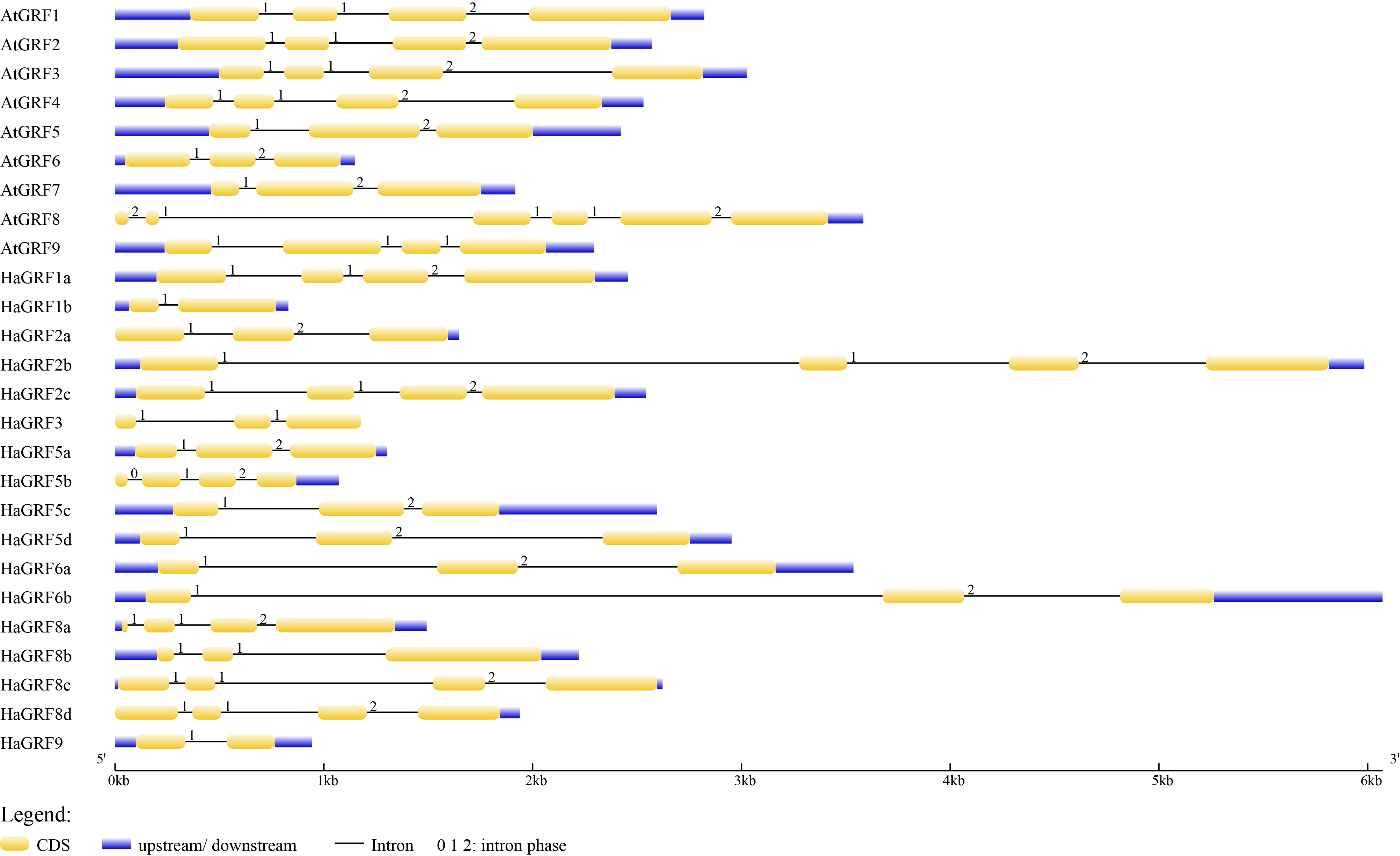

Supplement: Supplementary file 1 [file plants-14-03484-s001.zip › Figure S1.jpg]
